# Supplementary material for: Capturing transient states of heterodimeric ABC transporter TM287/288 by time-resolved small-angle X-ray scattering
Source: Biophys J. 2026 Apr 18;125(11):2648–56. doi: 10.1016/j.bpj.2026.04.016 (PMC13351893; doi:10.1016/j.bpj.2026.04.016)
Supplement: Document S1. Figures S1–S7 and Table S1 [file mmc1.pdf]

Biophysical Journal, Volume *125*

Supplemental information

Capturing transient states of heterodimeric ABC transporter TM287/288 by time-resolved small-angle X-ray scattering

Lea Schröder, Dario De Vecchis, Andrey Gruzinov, Lars V. Schäfer, Clement E. Blanchet, Markus A. Seeger, Henning Tidow, and Inokentijs Josts

Supplementary Information for:

Capturing transient states of heterodimeric ABC transporter TM287/288 by Time-Resolved Small-Angle X-ray Scattering

Lea Schröder[1,2], Dario De Vecchis[3,7], Andrey Gruzinov[4], Lars V. Schäfer[3], Clement E. Blanchet[4], Markus A. Seeger[5], Henning Tidow[1,2]*, and Inokentijs Josts[1,2,6]*

[1] The Hamburg Advanced Research Centre for Bioorganic Chemistry (HARBOR), Luruper Chaussee 149, D-22761 Hamburg, Germany

[2] Department of Chemistry, University of Hamburg, Luruper Chaussee 149, D-22761 Hamburg, Germany

[3] Center for Theoretical Chemistry, Ruhr University Bochum, Universitätsstr. 150, D-44801 Bochum, Germany

[4] European Molecular Biology Laboratory Hamburg Outstation c/o DESY, Notkestrasse 85, D-22607 Hamburg, Germany

[5] Institute of Medical Microbiology, University of Zurich, Zürich, Switzerland

[6] Newcastle University, Faculty of Medical Sciences, Newcastle University Biosciences Institute (NUBI), Framlington Place, Newcastle upon Tyne NE2 4HH, UK

[7] Present address: Univ. Grenoble Alpes, CNRS, CEA, IBS, France

* Corresponding authors:

Inokentijs Josts
Newcastle University, Faculty of Medical Sciences, Newcastle University Biosciences Institute (NUBI), Framlington Place, Newcastle upon Tyne NE2 4HH, UK
e-mail: Kesha.Josts@newcastle.ac.uk

Henning Tidow
University of Hamburg, The Hamburg Advanced Research Centre for Bioorganic Chemistry (HARBOR), Luruper Chaussee 149, D-22761 Hamburg, Germany
e-mail: henning.tidow@uni-hamburg.de

Supplementary figures

Suppl. Figure S1

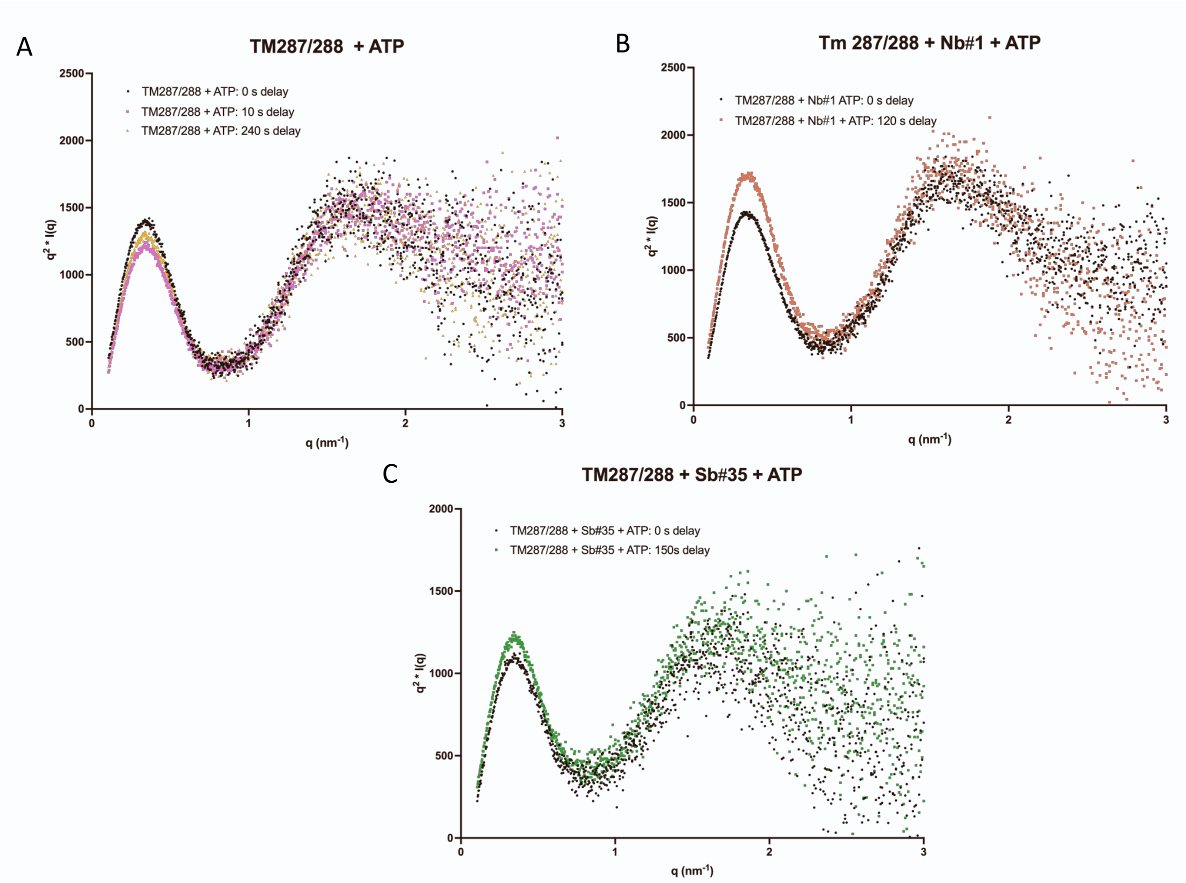

Kratky plots of representative experimental SAXS curves at selected time points. A) Three time points of TM287/288 binding ATP-Mg^{2+} representing evolution of conformational changes in the sample. B) Two time points showing changes in scattering of TM287/288 upon binding ATP and Nb#1. C) Two time points showing changes in scattering of TM287/288 upon binding ATP and Sb#35.

Suppl. Figure S2

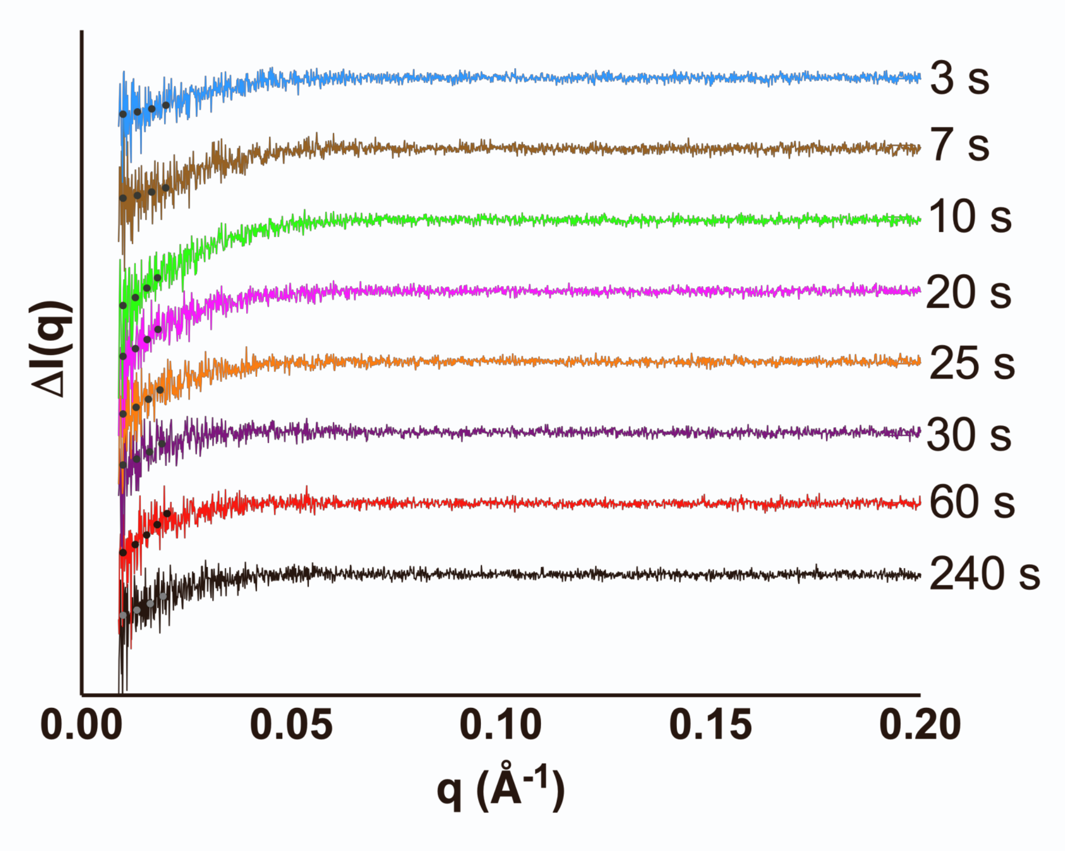

Time-resolved scattering difference curves show conformational changes in TM287/288 upon ATP binding. Stacked ΔI(q) curves showing changes in scattering signal over reaction time. Dotted lines emphasise changes in low-q region where Guinier fits were calculated.

Suppl. Figure S3

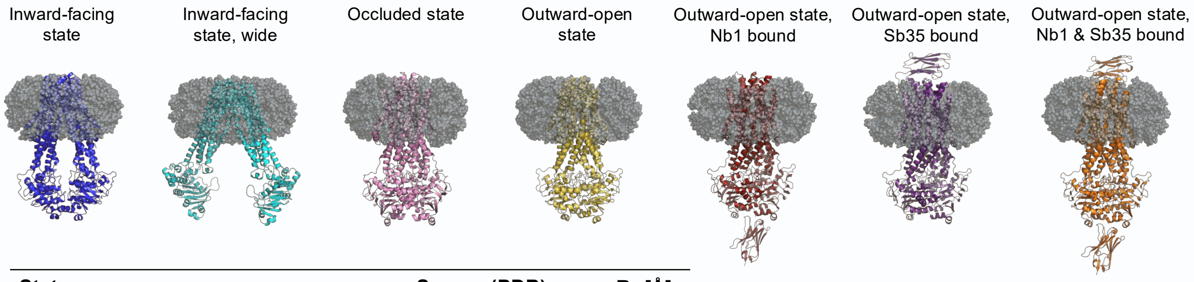

State	Source (PDB)	R_g [Å]
Inward-facing	4Q4A	44.2
Inward-facing, wide	6BL6	47.9
Occluded	modelled from: 4S0F, 7PR1, 6RAI	44.0
Outward-open	6QUZ	45.4
Outward-open, Nb1 bound	modelled from: 6QV1	48.5
Outward-open, Sb35 bound	modelled from: 6QUZ	46.0
Outward-open, Nb1 & Sb35 bound	modelled from: 6QV1, 6QUZ	48.9

Illustration of different conformational states of TM287/288 in detergent (DDM) micelles with R_g values indicated. PDB codes of structures or templates for models are given in the table. inward-facing state, blue / wide inward-facing apo state, cyan / occluded state, pink / outward-open state, yellow / outward-open, Nb1 bound state, red / outward-open Sb35-bond state, violet / outward-open Nb1 and Sb35-bound state, orange. Detergent micelles were added using CHARMM-GUI micelle builder (Cheng, Jo et al. 2013), and R_g values were calculated using CRYSOL (Svergun, Barberato et al. 1995).

Suppl. Figure S4

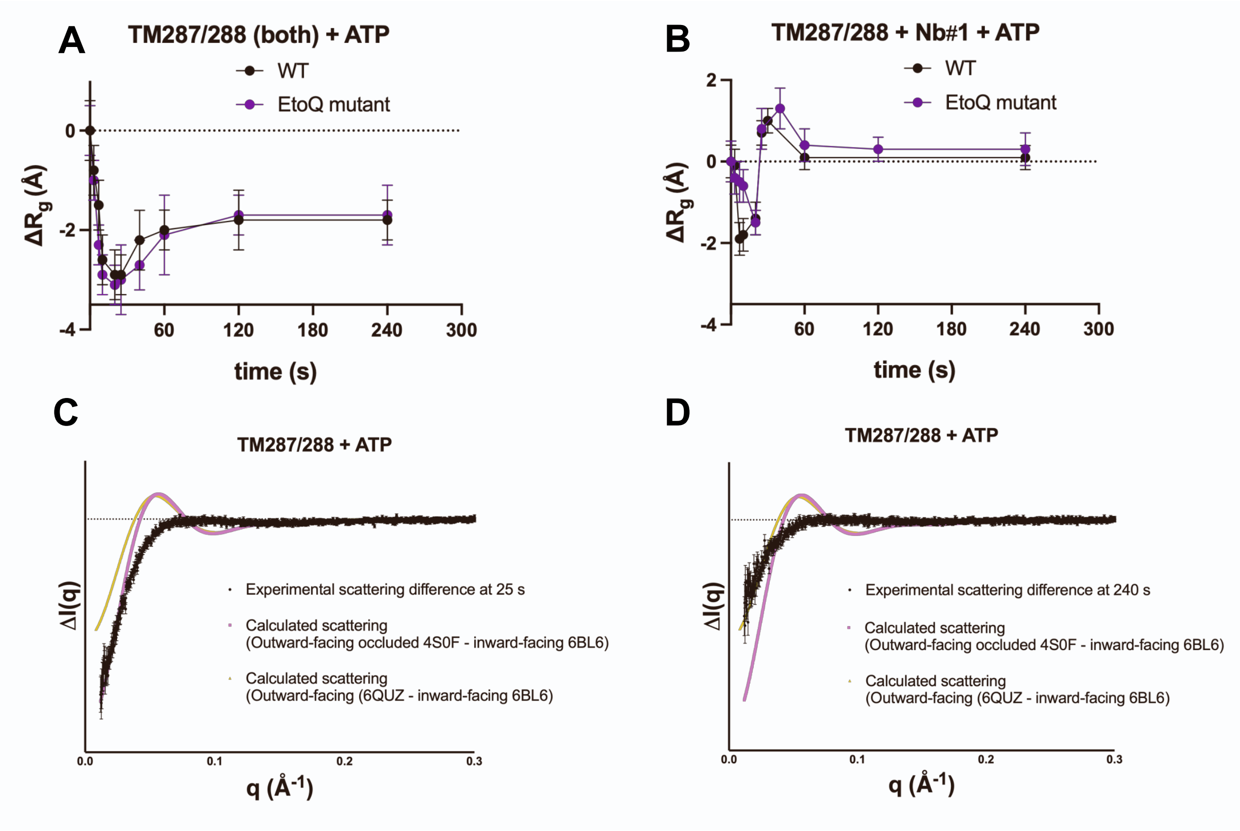

SF-TR-SAXS comparison of wt TM287/288 and its E/Q-mutant (E517Q). A) Apo proteins mixed with ATP-Mg^{2+}. B) TM287/288 variants premixed with Nb#1 before SF-mixed with ATP-Mg^{2+}. C) and D) Comparison of the 25 s time point (where the occluded state is thought to populate) and 240 s time point (where outward-open state is thought to populate) in our kinetic reaction with two calculated models of TM287/288. The χ^2 values for the fit between 25 s and Occ model is 13.8 versus 26.7 for OF model. At 240 s the χ^2 for Occ is 10 but 3.9 for OF model. The 240 s difference dataset is noisier and larger experimental uncertainties lead to lower χ^2 values.

Suppl. Figure S5

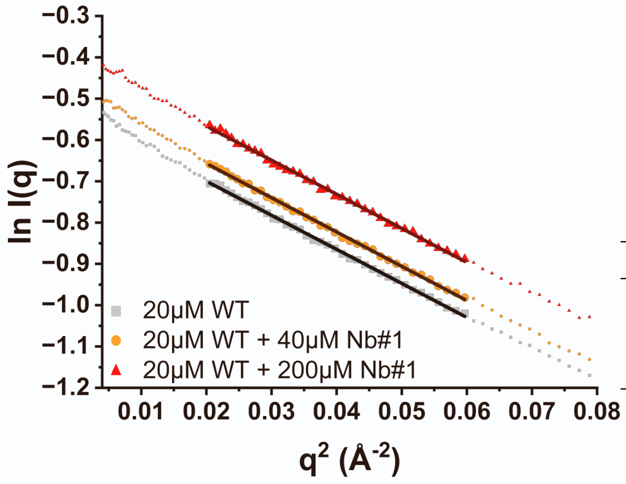

sample	slope Guinier plot	R_g [Å]
TM287/288 WT	-8.25 ± 0.05	49.7 ± 0.2
TM287/288 WT + 2x Nb#1	-8.30 ± 0.05	49.9 ± 0.2
TM287/288 WT + 10x Nb#1	-8.25 ± 0.07	49.7 ± 0.3

Guinier analysis of apo TM287/288 in absence and presence of Nb#1 indicate that Nb#1 is not binding to TM287/288 in the absence of Mg^{2+}-ATP.

Suppl. Figure S6

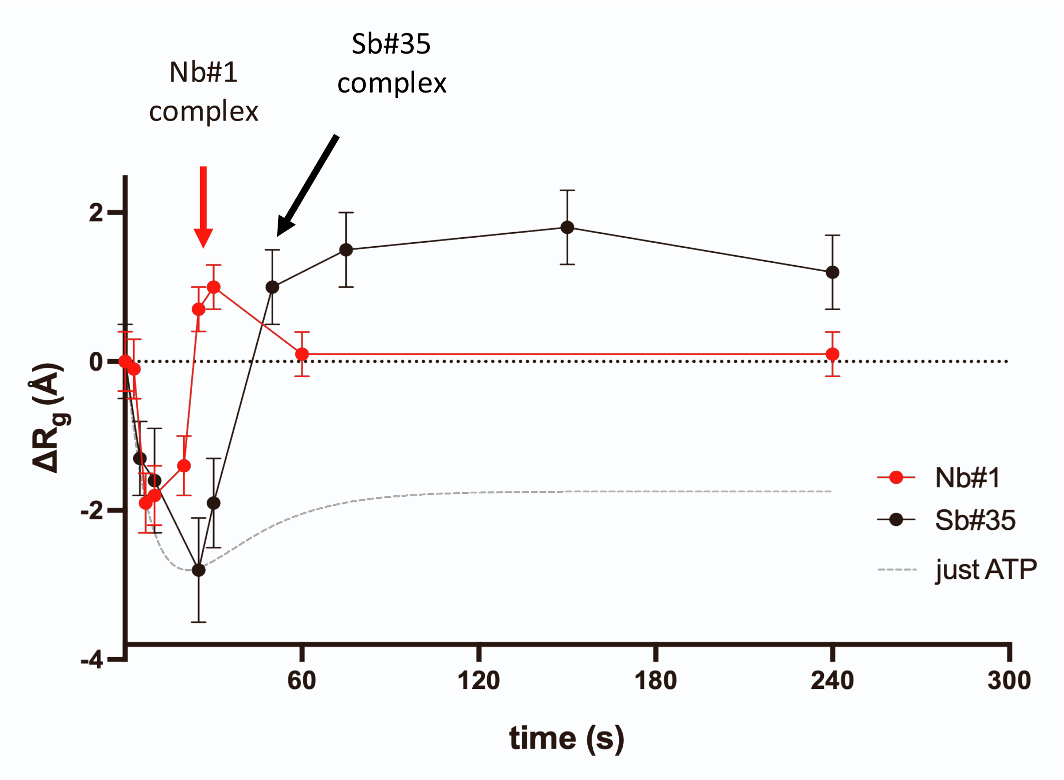

SF-TR-SAXS comparison of TM287/288 in complex with Nb#1 and Sb#35. SF-TR-SAXS data acquired for TM287/288 premixed with Nb#1 (A) or Sb#35 (B) before SF-mixed with ATP-Mg^{2+}. Nb#1 binds to the NBDs of TM287/288 in the occluded state while Sb#35 binds to the extracellular side only in the OF state.

Suppl. Figure S7

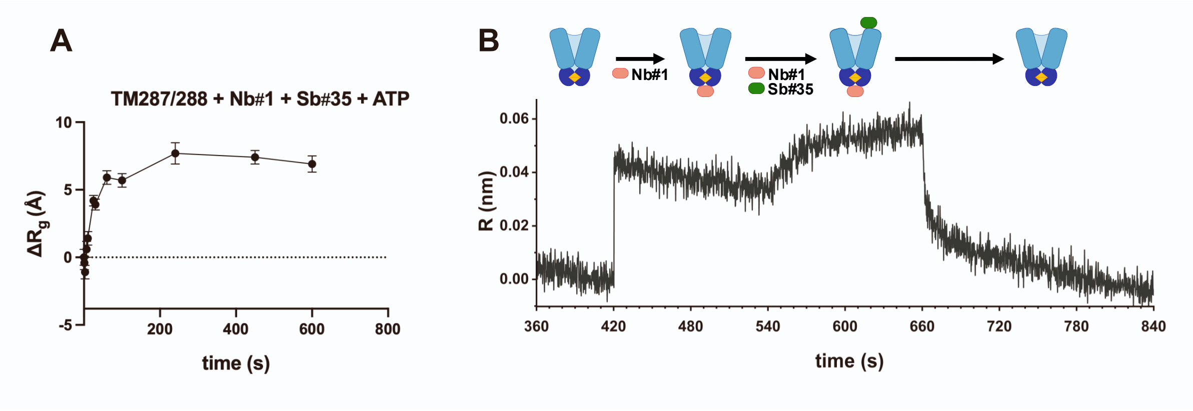

Simultaneous binding of both single-domain antibodies to TM287/288. A) SF-TR-SAXS data acquired for TM287/288 premixed with both Nb#1 and Sb#35 before SF-mixed with ATP-Mg^{2+}. B) BLI measurements showing association of Nb#1 and Sb#35 followed by their dissociation.

Suppl. Table 1

	K_m	v_{max}	k_{cat}
	mM	*nmol*$_{ATP}$*/min/mg*$_{TM287/288}$	*min*$^{-1}$
**wt**	0.059 ± 0.008	67.7 ± 1.5	9.02 ± 0.20
**wt 2x Nb1**	0.039 ± 0.005	45.6 ± 0.8	6.07 ± 0.11
**wt 10x Nb1**	0.066 ± 0.008	41.4 ± 0.9	5.52 ± 0.12
**wt 2x Sb35**	0.041 ± 0.005	49.5 ± 0.9	6.61 ± 0.12
**wt 10x Sb35**	0.048 ± 0.008	36.5 ± 0.9	4.87 ± 0.12
**E517Q**	0.038 ± 0.018	12.5 ± 0.8	1.67 ± 0.11

Suppl. Table S1: ATPase activity assays. Activity assays for TM287/288 were performed using the Baginski method at room temperature (Baginski, Epstein et al. 1975, Chifflet, Torriglia et al. 1988). K_m and k_{cat} were calculated from fitting the experimental data according to Michaelis-Menten. Standard errors were obtained from triplicate measurements.

References

Baginski, E. S., E. Epstein and B. Zak (1975). "Review of phosphate methodologies." Ann Clin Lab Sci **5**(5): 399-416.

Cheng, X., S. Jo, H. S. Lee, J. B. Klauda and W. Im (2013). "CHARMM-GUI micelle builder for pure/mixed micelle and protein/micelle complex systems." J Chem Inf Model **53**(8): 2171-2180.

Chifflet, S., A. Torriglia, R. Chiesa and S. Tolosa (1988). "A method for the determination of inorganic phosphate in the presence of labile organic phosphate and high concentrations of protein: application to lens ATPases." Anal Biochem **168**(1): 1-4.

Svergun, D. I., C. Barberato and M. H. J. Koch (1995). "CRYSOL - a Program to Evaluate X-ray Solution Scattering of Biological Macromolecules from Atomic Coordinates." J. Appl. Crystallogr. **28**: 768-773.
