# Supplementary material for: Capturing transient states of heterodimeric ABC transporter TM287/288 by time-resolved small-angle X-ray scattering
Source: Biophys J. 2026 Apr 18;125(11):2648–56. doi: 10.1016/j.bpj.2026.04.016 (PMC13351893; doi:10.1016/j.bpj.2026.04.016)
Supplement: Document S2. Article plus supplemental information [file mmc2.pdf]

Biophysical Journal
Article

Biophysical Society

Capturing transient states of heterodimeric ABC transporter TM287/288 by time-resolved small-angle X-ray scattering

Lea Schröder,[1,2] Dario De Vecchis,[3,7] Andrey Gruzinov,[4] Lars V. Schäfer,[3] Clement E. Blanchet,[4] Markus A. Seeger,[5] Henning Tidow,[1,2,*] and Inokentijs Josts[1,2,6,*]

[1]The Hamburg Advanced Research Centre for Bioorganic Chemistry (HARBOR), Luruper Chaussee 149, 22761 Hamburg, Germany; [2]Department of Chemistry, University of Hamburg, Luruper Chaussee 149, 22761 Hamburg, Germany; [3]Center for Theoretical Chemistry, Ruhr University Bochum, Universitätsstr. 150, 44801 Bochum, Germany; [4]European Molecular Biology Laboratory Hamburg Outstation c/o DESY, Notkestrasse 85, 22607 Hamburg, Germany; [5]Institute of Medical Microbiology, University of Zurich, Zürich, Switzerland; and [6]Newcastle University, Faculty of Medical Sciences, Newcastle University Biosciences Institute (NUBI), Framlington Place, Newcastle upon Tyne NE2 4HH, UK
[7]Present address: University Grenoble Alpes, CNRS, CEA, IBS, France

ABSTRACT Structures of the heterodimeric ATP-binding cassette (ABC) transporter TM287/288 have previously been determined in several states indicating large conformational changes during its reaction cycle. However, for a complete description of the cycle, transient states (such as an occluded state) are still missing, as they are difficult to capture with static structural biology techniques. Here, we investigate the kinetics and accompanying structural changes in the course of the reaction cycle of full-length TM287/288 using time-resolved small-angle X-ray scattering initiated by stopped-flow mixing. The use of active-site mutants and state-specific sybodies/nanobodies enabled us to dissect the temporal events involved in the ATP-driven conformational cycle of TM287/288 and reveal a transient occluded state of this ABC transporter.

SIGNIFICANCE ATP-binding cassette (ABC) transporters are essential molecular machines that use the power of ATP binding and hydrolysis to move substances across cellular membranes. Here, we use time-resolved small-angle X-ray scattering combined with conformation-specific nanobodies to directly capture and assign different states to the heterodimeric ABC transporter TM287/288 in solution. Our findings advance our understanding of the ABC transporter and demonstrate a broadly applicable method for dissecting the different molecular states in membrane proteins on a second-to-minute timescale.

INTRODUCTION

ATP-binding cassette (ABC) transporters are ubiquitous integral membrane proteins found across all domains of life.[1–3] These transporters mediate the ATP-dependent translocation of a wide range of substrates—including ions, lipids, peptides, and large organic molecules—across biological membranes.[4–7] Based on their transport directionality, ABC transporters are generally classified as importers, exporters, or extruders.[8] They operate as homo- or heterodimers, coupling ATP-Mg^{2+} binding, ATP hydrolysis, and ADP/P_i release to substrate translocation via a mechanochemical mechanism.[9]

ABC transporters share a conserved architecture composed of two transmembrane domains (TMDs) and two nucleotide-binding domains (NBDs). The TMDs define substrate specificity and form the translocation pathway, whereas the NBDs supply the free energy required for transport via ATP binding and hydrolysis. Early mechanistic descriptions framed this coupling in terms of a nucleotide-driven conformational "power stroke," in which ATP-induced NBD dimerization promotes large-scale rearrangements of the TMDs.[9–11] Subsequent biochemical, structural, and spectroscopic studies have demonstrated that this process is better described as a series of coordinated and reversible conformational equilibria linking nucleotide occupancy at the NBDs to

Submitted November 6, 2025, and accepted for publication April 16, 2026.

*Correspondence: henning.tidow@uni-hamburg.de or kesha.josts@newcastle.ac.uk

Editor: Merritt Maduke.

https://doi.org/10.1016/j.bpj.2026.04.016

Crown Copyright © 2026 Published by Elsevier Inc. on behalf of Biophysical Society.
This is an open access article under the CC BY license (http://creativecommons.org/licenses/by/4.0/).

alternating-access transitions of the TMDs rather than a single discrete mechanical step.[12–14]

In ABC exporters, the transport cycle is commonly described by an alternating-access mechanism in which the resting inward-facing (IF) conformation with separated NBDs transitions to an outward-facing (OF) conformation upon ATP-induced NBD dimerization, followed by ATP hydrolysis, NBD disengagement, and return to the IF state.[14] Although still debated, ATP binding alone seems to be sufficient for the IF-to-OF transition, while ATP hydrolysis and phosphate release will subsequently reset the transporter to the IF state.[15–17] Numerous structures of ABC transporters have been determined in various conformational states (IF/occluded [Occ]/OF) providing distinct snapshots of the conformational cycle.[8,18–22] In type IV ABC transporters, communication between NBDs and TMDs is mediated via large intracellular domains containing "coupling helices."[3] Recent integrative structural, biochemical, and computational studies have further refined this model, providing detailed insight into the temporal order of nucleotide binding, hydrolysis, and conformational transitions in type IV ABC transporters.[15,17,23,24]

Importantly, not all ABC transporters possess two catalytically equivalent ATPase sites. Many heterodimeric ABC transporters exhibit intrinsic asymmetry, with one consensus site capable of ATP hydrolysis and one degenerate site that binds but does not hydrolyze ATP.[8] TM287/288, a heterodimeric ABC exporter from *Thermotoga maritima*, exemplifies this class. It contains one catalytically competent ATP-binding site and one degenerate site, resulting in asymmetric nucleotide processing during the transport cycle.[25] Although its native substrate is unknown, TM287/288 has been shown to transport the anticancer drug daunomycin as well as Hoechst 33342.[26] TM287/288 has been structurally characterized in multiple nucleotide- and conformation-dependent states, making it a well-established model for dissecting the mechanistic consequences of ATPase asymmetry in ABC exporters.[26–28]

Recently, two conformation-specific single-domain antibodies were developed that selectively recognize and stabilize the OF conformation of TM287/288, thereby inhibiting its transport activity.[28] Sybody Sb#35 binds to the extracellular wing region of the transporter, whereas nanobody Nb#1 targets the closed NBD dimer interface from the cytosolic side with an epitope spanning both TM287 and TM288. Binding of both antibodies is ATP dependent and results in inhibition of ATPase activity, providing powerful tools for selectively trapping defined functional states along the transport cycle.

Time-resolved small-angle X-ray scattering (SAXS) with stopped-flow mixing (SF-TR-SAXS) is a solution-based technique capable of capturing protein conformational changes on the millisecond timescale.[29] SF-TR-SAXS has been successfully applied to study protein folding reactions and ligand-induced quaternary structural transitions.[30–33] We previously employed SF-TR-SAXS to resolve the kinetics of NBD dimerization and dissociation in both isolated NBDs and full-length MsbA reconstituted in lipid nanodiscs, demonstrating its suitability for dissecting transporter conformational dynamics in real time.[34]

In the present study, we use SF-TR-SAXS to investigate the conformational dynamics of the heterodimeric ABC exporter TM287/288. By exploiting conformation-specific single-domain antibodies to selectively stabilize distinct functional states, we assign time-resolved SAXS signatures to discrete steps of the transport cycle and identify transient intermediates that are not readily accessible by static structural methods.

Collectively, our data provide a kinetic and structural dissection of the conformational transitions underlying the functional cycle of the heterodimeric ABC transporter TM287/288, placing these observations within the context of previously established mechanistic models for type IV ABC exporters and extending them through direct experimental characterization of transient intermediates.

MATERIALS AND METHODS

Expression and purification of full-length TM287/288

The plasmid encoding for the heterodimeric ABC transporter TM287/288 of *T. maritima* has previously been described.[26] The expression vector is pBXNH3L. Expression and purification were adapted from Hutter et al.[28]: Transformed *Escherichia coli* MC1061 cells were grown in terrific broth (TB) medium supplemented with 100 μg/mL ampicillin at 37°C for 1.5 h and further grown at 30°C until an optical density 600 (OD_{600}) of 1.5 was reached. Expression was then induced with 0.0017% (w/v) L-arabinose. After 5 h, cells were harvested. For membrane preparation, cells were disrupted in lysis buffer (20 mM Tris, 200 mM NaCl, and 10% [v/v] glycerol, pH 7.5) supplemented with lysozyme, DNase, and protease inhibitor. For solubilization, the membrane pellet was then resuspended in lysis buffer and supplemented with 1% (w/v) dodecylmaltoside (DDM). Membranes were solubilized for 2 h at 4°C. After addition of 30 mM imidazole, the supernatant was applied onto an Ni-NTA column. The column was washed with 50 mM imidazole, 200 mM NaCl, 10% glycerol, and 0.03% DDM at pH 7.5. TM287/288 eluted with 200 mM imidazole, 200 mM NaCl, 10% glycerol, and 0.03% DDM at pH 7.5. The buffer was exchanged for 20 mM Tris, 150 mM NaCl, and 0.03% DDM (pH 7.5), and size exclusion was performed using a Superose 6 Increase 10/300 column.

Compared to its various PDB structures the TM287/288 protein contains an additional N-terminal His-tag and linker sequence: MHHHHHHHHHHLEVLFQGPSGSGGGGGS.

Expression and purification of nanobody Nb#1 and sybody Sb#35

The plasmids encoding for nanobody Nb#1 and sybody Sb#35 have previously been described.[28] The expression vector is pSBinit. Transformed *E. coli* MC1061 cells were

grown in TB medium supplemented with 25 μg/mL chloramphenicol at 37°C and 100 rpm for 2 h and further grown at 25°C until an OD_{600} of 1.5 was reached. Expression was then induced with 0.02% (w/v) L-arabinose. After incubation overnight, cells were harvested. Cells were disrupted in lysis buffer (20 mM Tris, 200 mM NaCl, and 10% [v/v] glycerol, pH 7.5) supplemented with lysozyme, DNase, and protease inhibitor and afterward centrifuged. The supernatant was supplemented with 30 mM imidazole and applied onto a Ni-NTA column. The column was washed with 50 mM imidazole, 200 mM NaCl, and 10% glycerol at pH 7.5, and Nb1/Sb35 finally eluted with 200 mM imidazole, 200 mM NaCl, 10% glycerol, and 0.03% DDM at pH 7.5. The buffer was exchanged for 20 mM Tris and 150 mM NaCl (pH 7.5) and size exclusion performed using a HiLoad 16/600 Superdex 75 column.

Compared to the PDB model 6qv1, the Nb#1 protein contains the additional residues GRAGEQKLISEEDLNSAV DHHHHHH. Compared to the PDB models 6quz and 6qv0, the Sb#35 protein contains the additional residues AGRAGEQKLISEEDLNSAVDHHHHHH.

(Stopped-flow time-resolved) small-angle X-ray scattering experiments

All synchrotron SAXS data were collected at beamline P12 operated by EMBL Hamburg at the PETRA III storage ring (DESY, Hamburg, Germany).[35] For batch measurements, TM287/288 was dialyzed against Tris-buffered saline (TBS) buffer (20 mM Tris, 150 mM NaCl, pH 7.5) + 0.03% DDM. TM287/288 was measured at a final concentration of 20 μM. Nb#1 in TBS + 0.03% DDM was added in either 2× molar excess (40 μM) or 10× molar excess (200 μM). All batch measurements were performed at 20°C, with data collected on the Pilatus 6M detector. A total of 10 frames with 100-ms exposure per frame were collected for all the protein samples, with buffer frames collected before and after each protein measurement. Data were averaged and background subtracted against the appropriate buffers using automatic procedures on the beamline.

The following experiments were performed in 20 mM HEPES, pH7.6, 200 mM NaCl, 5 mM $MgCl_2$. For the stopped-flow time-resolved SAXS experiments, TM287/288 purified in DDM (~20 μM) was mixed with Mg^{2+}-ATP (1 mM) using a stopped-flow device, simultaneously injecting 60 μL of each component with a flow-rate of 2 mL/s resulting in a dead-time of 5.0 ms (Bio-logic, Seyssinet-Pariset, France). Nb#1 and Sb#35 were loaded with TM287/288 in 2× excess in one of the syringes for the kinetic experiments. All stopped-flow measurements were performed at room temperature (RT) with data collected on the Eiger 4M detector with a sample to detector distance of 3 m. The SAXS experiments were carried out with a wavelength of 1.24 Å (10 keV), transmission was set to 30% (beam flux ≈ 2.10^{12} photons/s), and X-ray exposure time was set to 25 ms. 40 frames were acquired per injection. Kinetics were captured by adjusting the delay between the sample mixing and the acquisition of the scattering data. The 40 frames collected for each injections were compared using CorMap[36] (Franke et al.) and statistically similar frames were averaged.

SAXS data were analyzed using ATSAS.[37] Radius of gyration (R_g) values were calculated using Primus with errors obtained from the Guinier fit.

Modeling of TM287/288 Occ-state structures

TM287/288 structural models were built with Modeller[38] using available templates. Residues 2–569 for TM287 and residues 22–591 for TM288 were considered. Nucleotides were not modeled. Accession numbers are available for the IF state (PDB: 4Q4A), wide IF state (PDB: 6BL6), (OF) Occ state (PDB: 4S0F, 7PR1, and 6RAI), outward-open state (PDB: 6QUZ), and outward-open state with Nb#1/Sb#35 bound (PDB: 6QUZ).

Modeling and data analysis

Models of DDM detergent-micelle-embedded TM287/288 in different conformations were built using CHARMM-GUI micelle builder with 170 β-DDM molecules,[39] and CRYSOL[40] was used to calculate the R_g of the models and theoretical scattering curves.

Activity assays

TM287/288 activity was measured performing the Baginski assay[41,42] as previously described.[43] Five micrograms of TM287/288 in activity assay buffer (20 mM Tris, 150 mM NaCl, 5 mM $MgCl_2$, 0.03% DDM) were incubated with 0/0.05/0.1/0.2/0.35/0.5/0.75/1/2/4 mM ATP in a total volume of 50 μL for 20 min at RT (final TM287/288 concentration, 0.75 μM). Addition of 50 μL of freshly prepared ascorbic acid solution (140 mM ascorbic acid, 0.5 M HCl, 0.1% SDS, 5 mM ammonium heptamolybdate) stopped the reaction. After 10-min incubation at RT, 75 mL of sodium citrate solution (2% sodium citrate, 2% sodium metaarsenite, 2% acetic acid) were added to stabilize the color. After ~45 min, absorbance at 860 nm was read using a Tecan Spark 20 M. To assess how Nb#1 and Sb#35 affect activity, they were added to wild-type (WT) TM287/288 in 2× and 10× molar excess analogous to the time-resolved SAXS experiments. The activity assays were then performed as described above. All experiments were performed as triplicates and, for comparability to time-resolved SAXS, at RT.

Biolayer interferometry

TM287/288 (WT) was biotinylated for biolayer interferometry (BLI) experiments. The biotinylation reaction

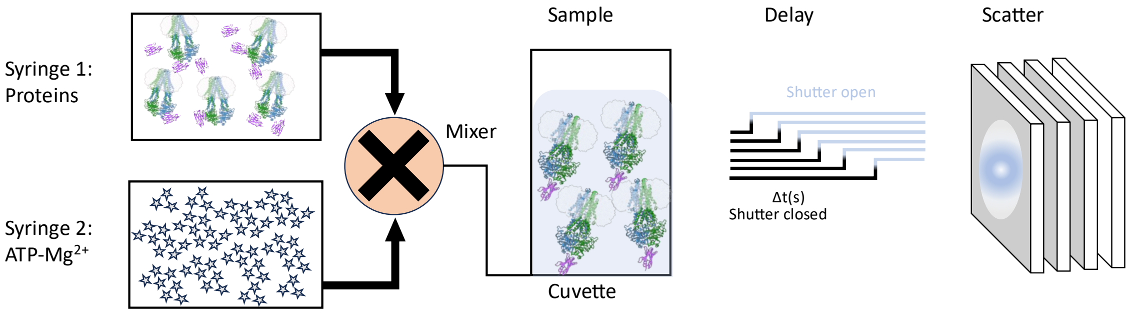

Figure 1. Schematic setup for time-resolved SAXS measurements with TM287/288 and single-domain antibodies (Nb#1/Sb#35). TM287/288 (in absence or presence of Nb#1/Sb#35) in syringe 1 was mixed with ATP-Mg^{2+} in syringe 2, and SAXS data were acquired after various time delays ranging from 0 ms to 240 s. The first measurement (zero delay) consists of the acquisition of 40 frames with 25-ms exposure each. Therefore, the first time point in our experiments is an average of all frames between 0 and 1 s. Subsequent delays are also averages of 1,000-ms frames from the time of shutter opening.

was performed mixing TM287/288 WT in 137 mM NaCl, 2.7 mM KCl, 10 mM Na_2HPO_4, 1.8 mM KH_2PO_4, and 0.03% DDM pH 6.5 with EZ-link sulfo-NHS-LC-LC-biotin in DMSO in a 1:5 molar ratio. The mixture was incubated at 4°C for 24 h. Excess biotin was removed and the buffer exchanged for 20 mM Tris, 150 mM NaCl, and 0.03% DDM pH 7.5. BLI measurements were performed at 25°C using the Octet RED96 system by Sartorius. For all steps, buffer containing 20 mM Tris, 150 mM NaCl, 5 mM $MgCl_2$, 1 mM ATP, and 0.03% DDM (pH 7.5) was used. Streptavidin sensors were pre-equilibrated in buffer for ~30 min. A buffer baseline was measured for 60 s followed by immobilization of 3 μg/mL TM287/288 WT (0 μg/mL for reference) onto the sensors for 240 s. After a second baseline (120 s), the association of 40 μM Nb#1 was recorded for 120 s followed by a second association phase of 120 s with 40 μM Nb#1 and 40 μM Sb#35. Buffer was used to dissociate the analytes for 500 s.

RESULTS AND DISCUSSION

Transitions between different conformational states of TM287/288 can be investigated by SF-TR-SAXS

Using the stopped-flow setup installed at the P12 BioSAXS beamline at EMBL, Hamburg,[35] we mixed apo-TM287/288 purified in DDM (20 μM) with Mg^{2+}-ATP (1 mM) and recorded SAXS profiles at RT at regular intervals (from 0 s to 4 min) (Figure 1). Mixing of Mg^{2+}-ATP with purified TM287/288 led to a decrease in the R_g of TM287/288 after 1 s, reaching a minimum at ~20 s (Figure 2A). After 30 s, the R_g increases slightly and plateaus until our latest time point of 240 s without returning back to the original size. Kratky plots of representative experimental SAXS curves at selected time points are shown in Figure S1. Scattering difference curves show that conformational changes occur in the q region 0.015–0.05 $Å^{-1}$ implying large-scale structural changes in TM287/288 upon ATP binding (Figure S2). Our previous work with the ABC exporter MsbA in nanodiscs has concluded that the initial reduction in protein size can be attributed to the dimerization of the NBDs and the formation of an Occ transporter state.[34] Therefore, we interpret the phase of initial R_g compaction in TM287/288 as ATP-induced dimerization of the NBDs leading to an Occ state after 20–30 s. Such an Occ state of TM287/288 has not been experimentally shown before, but simulations of the catalytic cycle of TM287/288 showed that this intermediate does form *in silico*.[44,45] Therefore, we modeled this state using PCAT1 (PDB: 4S0F[20]), CtAtm1 (PDB: 7PR1[19]), and TmrAB (PDB: 6RAI[24]) ABC transporters in (OF) Occ conformation as templates (Figures 2

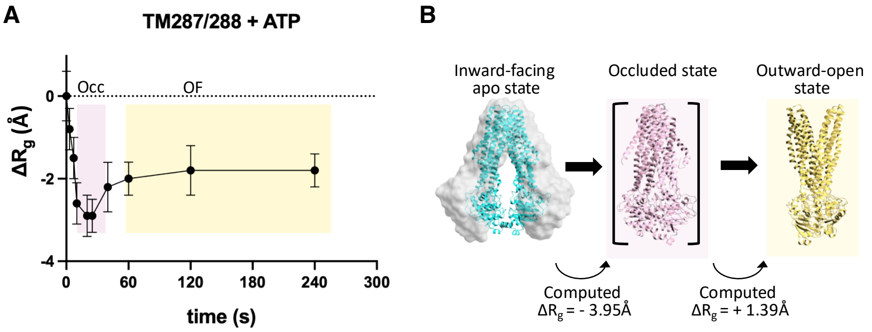

Figure 2. ATP-driven conformational changes of TM287/288 can be followed by TR-SAXS. (A) SAXS data acquired after mixing and various delays were used to calculate changes in radius of gyration (R_g) with errors obtained from the Guinier fit. The dashed line corresponds to the starting state. (B) Conformational changes of TM287/288 models that were assigned to the phases in the TR-SAXS profile. The gray envelope corresponds to a wide-open apo model. R_g values were calculated with CRYSOL.

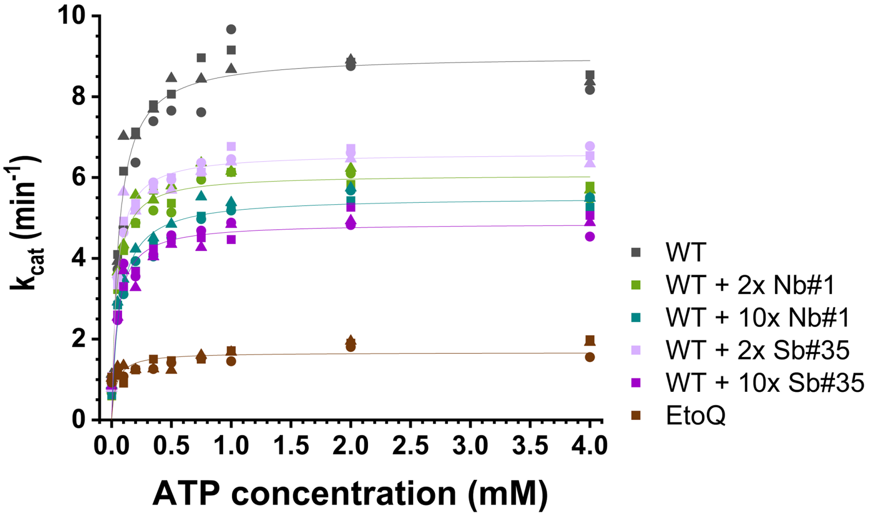

Figure 3. Activity assays and binding data of TM287/288 and conformation-specific single-domain antibodies. Activity assays showing decrease in activity of TM287/288 when bound to conformation-specific single-domain antibodies Nb#1 or Sb#35. All assays were performed as triplicates represented by the different symbols for each data point. The data were then fitted to the Michaelis-Menten equation.

and S3). The observed change in R_g (ΔR_g) in our SAXS data is consistent with the calculated R_g change based on structural models of TM287/288 in the different conformational states (Figure 2B). Additionally, comparison of calculated states of TM287/288 (Occ and OF) against our experimental scattering difference curves at 25 s and 240 s support these findings (Figures S4C and S4D).

It should be noted that the assignment of the Occ state (as well as other conformational states) is based on agreement between experimental and calculated R_g changes, making use of existing structures in different conformational states. While this indirect assignment does not rule out alternative conformations leading to similar R_g changes, our assignment is supported by a wealth of prior ABC transporter studies showing that ATP binding to the apo proteins leads to NBD dimerization and thus compaction of the transporter.

To investigate whether the increase in R_g after 20 s could be a consequence of ATP hydrolysis and subsequent dissociation of the NBDs, we used a catalytically inactive E/Q mutant (TM288$_{E517Q}$) as comparison. This E/Q mutant has been shown not to hydrolyze ATP within the time frame of this experiment (half-life >30min[28]), and this was further confirmed by our ATP-hydrolysis activity assays (Figure 3). The TR-SAXS kinetic profile of the purified TM287/288 E/Q mutant is almost identical to that of the WT transporter (Figure S4A), indicating that ATP hydrolysis and resulting conformational changes can be ignored in our further analyses. Thus, our analysis covers the conformational changes from IF (start) to OF (end) without return.

This is further supported by the results of our activity assays, which show that ATP is hydrolyzed by the WT protein at RT (Figure 3). However, hydrolysis is slow at RT (k_{cat} = 9 min^{-1}/0.15 s^{-1}) because TM287/288 originates from *T. maritima* which has its temperature optimum around 80°C. Thus, we attribute the second phase of increase in R_g beginning around 30 s to the transition between Occ state and OF state of the transporter (Figure 2B).

The conditions required for an ABC transporter to adopt its OF conformation—the high-energy state of its transport cycle—remain under debate. Some studies argue that ATP hydrolysis is essential for OF formation,[46,47] while others report that ATP binding alone suffices for the IF-to-OF transition.[15–17,48] Our SF-TR-SAXS data support the latter, indicating that ATP binding alone drives this conformational change, consistent with the ATP-switch model.[10]

In order to quantify the changes in R_g magnitude during the TM287/288 conformational cycle, we generated models of TM287/288 in DDM micelles based on available structures and homology models in various conformational states (Figure S3). We then calculated R_g values for these models using CRYSOL[40] (see table in Figure S3), calculated ΔR_g values between different states, and compared these ΔR_g values to our experimental TR-SAXS data. We compared ΔR_g rather than absolute R_g values in order to cancel out differences due to different protein constructs (e.g., His-tag and linker). Using the IF-Occ-OF transition of TM287/288 shown in Figure 2 as example, the R_g values change from 47.9 Å (IF starting structure) to 44.0 Å (Occ transition state) to 45.4 Å (OF state). The corresponding ΔR_g values of −3.9 and −2.5 Å agree well with our observed TR-SAXS data (Figure 2). The observed SAXS trace also supports the assumption of a wider open IF starting state than the current crystal structures of TM287/288 as a more closed IF starting state (such as PDB: 4Q4A) would not fit the data well. A closed IF starting state would result in a calculated ΔR_g of −0.2 Å and thus not agree with our observed experimental data (Figures 2 and S3). Our kinetic SAXS data reveal that TM287/288 undergoes a two-step conformational change upon nucleotide

addition: a rapid compaction phase leading to a minimal R_g around 20–30 s, followed by a slower expansion phase that plateaus between 2 and 4 min. These observations offer mechanistic insight into the sequential formation of an Occ intermediate and its transition to the OF state.

Conformational trapping of TM287/288 using state-specific single-domain antibodies

Recently, two different single-domain antibodies were generated that bind TM287/288 in the presence of ATP and inhibit its ATPase activity. In the crystal structures, both nanobodies bind the transporter in the OF state, with either nanobody Nb#1 bound to the bottom of the closed NBD dimer (PDB: 6QV1) or sybody Sb#35 bound to the top of an extracellular wing (PDB: 6QUZ).[28] They showed that Nb#1 and Sb#35 reduce ATPase activity by about 30% and 70%, respectively, at 10× molar excess,[28] which agrees well with our activity data shown in Figure 3.

We sought to use these conformation-specific nanobodies as markers of different TM287/288 states in our TR-SAXS experiments. In order to investigate their binding kinetics and the associated conformational changes, we mixed TM287/288 with either Nb#1 or Sb#35 and then added Mg^{2+}-ATP via stopped-flow mixing followed by SAXS profile acquisition. In previous studies it was shown that both single-domain nanobodies (Nb#1/Sb#35) bind preferentially to ATP-bound TM287/288 with some weak-affinity binding of Nb#1 also to apo TM287/288 at high concentrations.[28,49] Our static SAXS data, however, indicate no binding of Nb#1 to apo TM287/288 in the absence of ATP (Figure S5), thus we assume that their binding epitopes will only become accessible after ATP-induced conformational changes of the transporter.

For Nb#1, which binds at the interface of the dimerized NBDs, we observed a decrease in R_g during the first 20 s, which is attributed to the formation of an Occ state of TM287/288. This step is followed by a rapid increase in R_g, peaking around 45 s, which we associate with the binding of Nb#1 to the closed NBD dimer and a subsequent decrease in R_g over 15 s. No further change in R_g is observed up to the measured time point of 240 s (Figure 4A).

In the case of Sb#35, our TR-SAXS data show a similar overall trend with initial decrease in the R_g of the transporter, followed by subsequent increase in R_g but at a later time than with Nb#1 (Figure 4B). The plateau reached from 80 s shows larger R_g values than calculated from the model, most likely caused by minor (radiation-induced) aggregation of excess Sb#35.

Comparing the timescales of R_g changes in the presence of nanobody/sybody with those of apo TM287/288 with Mg^{2+}-ATP alone, it is evident that Nb#1 already binds to the Occ state of TM287/288 (to NBDs), while Sb#35 only binds to the OF state (from extracellular side). This agrees with the fact that the binding site for Nb#1, the closed NBD dimer, is formed in the Occ transporter conformation and is already accessible to the Nb#1. In contrast, the binding site for Sb#35, which is formed by the extracellular wing of TM287/288, only becomes accessible after transition of the transporter from the Occ state to the OF state. This explains the delayed rise in R_g from Sb#35 association during our mixing experiments (Figure S6).

The binding kinetics of the nanobodies had already been determined previously by surface plasmon resonance (SPR) (Sb#35 $k_{on} = 1.43 \times 10^4\ M^{-1}\ s^{-1}$, $k_{off} = 1.57 \times 10^{-3}\ s^{-1}$/Nb#1 $k_{on} = 3.41 \times 10^5\ M^{-1}\ s^{-1}$, $k_{off} = 6.28 \times 10^{-2}\ s^{-1}$).[28] However, binding kinetics of the nanobodies are unlikely to play a major role in this TR-SAXS setup, because the association phase of binding is very fast due to the high Nb/Sb concentrations (20 μM; ~1,000 × K_d). The off rate would only play a role under non-equilibrium situations (i.e., if free nanobodies are removed from the system), which is not the case in our setup.

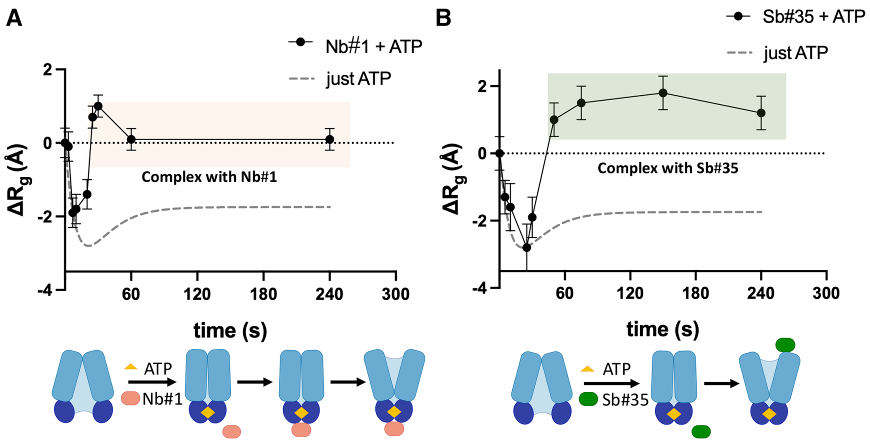

Figure 4. Using single-domain antibodies as conformational markers during TM287/288 turnover. SF-TR-SAXS data acquired for TM287/288 (20 μM) premixed with Nb#1 (A) or Sb#35 (B) (40 μM) before being SF mixed with ATP-Mg^{2+} (1 mM). Nb#1 binds to the NBDs of TM287/288 in the Occ state, while Sb#35 binds to the extracellular side only in the OF state.

An unresolved question concerns the "overshoot" around 45 s observed for the TM287/288+Nb#1 complex. This overshoot, which is absent in the complex with Sb#35 (Figure S6), could be explained by a two-step association mechanism of Nb#1 with changes in the NBD dimer interface.

Additionally, we could further show that both single-domain antibodies (Nb#1/Sb#35) can bind to TM287/288 simultaneously after mixing with Mg^{2+}-ATP, resulting in a large increase in R_g, consistent with our model of the ternary state (Figure S7A). The calculated R_g of the ternary state (TM287/288 + Nb#1 + Sb#35) is 48.9 Å (Figure S3). The simultaneous binding of both single-domain antibodies to TM287/288 is further supported by biolayer interferometry (BLI) data (Figure S7B). In this experiment, we immobilized biotinylated TM287/288 to the BLI sensor and measured binding to Nb#1 in the first step, resulting in a rapid signal increase (large k_{on}). In the second step, we further added Sb#35 and observed an additional signal increase (with slower k_{on}) indicating simultaneous binding of both single-domain antibodies. The differences in association kinetics of Nb#1 and Sb#35 to TM287/288 in the ternary complex agree with those previously observed for the binary complexes (see above).[28]

CONCLUSION

Altogether, our SF-TR-SAXS experiments provide a detailed mechanistic insight into the structural kinetics of ATP-driven conformations of the ABC transporter TM287/288. By observing changes in the transporter R_g, we can track transitions between its catalytic states on the second-to-minute timescale. To validate our findings, we make use of conformation-selective single-domain antibodies as temporal markers of different structural states in solution. With the advent of *de novo* binder designs targeting different protein conformations using machine-learning methods, techniques such as TR-SAXS can provide detailed structural kinetic insights into the molecular function of numerous proteins.

Upon rapid mixing with Mg^{2+}-ATP, we observed an immediate decrease in R_g, reaching a minimum within ~20–30 s. This compaction is consistent with ATP-driven dimerization of the NBDs and formation of an Occ state, which until now had only been predicted by simulations for TM287/288. Whether this Occ state resembles a fully Occ state or an OF-Occ state as previously shown for other ABC transporters[19,20,24] cannot be determined using this setup as their R_g values are very similar. Importantly, the subsequent partial re-expansion in R_g suggests a transition from the Occ to the OF state. This is in agreement with spontaneous large-scale conformational transitions from IF via Occ to OF conformation that have been observed by molecular dynamics (MD) simulations.[45] This two-step progression, unaffected in a catalytically inactive E/Q mutant, confirms that SF-TR-SAXS can resolve nucleotide-dependent conformational changes independent of hydrolysis within minutes. Comparison with calculated R_g values from structural models further supports the assignment of each state.

In addition, we exploited state-specific nanobodies (Nb#1 and Sb#35) as conformational traps. Their distinct binding kinetics and SAXS profiles reinforce the detection of the Occ and OF states. The ability to capture simultaneous binding of both nanobodies highlights the sequential accessibility of these conformations.

Interestingly, the conformation-specific nanobodies do not accelerate the formation of the OF-Occ or OF-open conformations. This may be explained by the "forward-only" reaction coordinate, meaning that, once ATP is bound, the reaction cannot reverse. In addition, it proves that nanobodies/sybodies do not artificially push the target protein into a new conformation but instead only stabilize a conformation once it is formed (= conformational trapping).

Together, these results demonstrate that SF-TR-SAXS is a powerful tool to resolve short-lived intermediates that are central to the transport mechanism. By combining rapid mixing with high-resolution structural modeling and conformationally selective nanobodies, we establish a robust framework to characterize dynamic states in ABC transporters and other complex membrane proteins.

DATA AVAILABILITY

- Representative SAXS data have been deposited at the SASBDB at www.sasbdb.org (SASBDB: SASDZ64, SASDZ74, and SASDZ84).
- Data for all time points are available for download from https://www.sasbdb.org/data/SASDZ64/ for the ATP-binding dataset, https://www.sasbdb.org/data/SASDZ74/ for the ATP and Nb_TM#1 dataset, and https://www.sasbdb.org/data/SASDZ84 for the ATP and Sb#35 dataset.
- All SAXS data processing and analysis were performed using the ATSAS suite.[37]
- Visualization and image preparation were performed with PyMOL, Excel, Adobe Illustrator, GraphPad Prism, and PowerPoint.

ACKNOWLEDGMENTS

We are grateful to the staff at the beamline P12 (EMBL, Hamburg) and acknowledge access to the Sample Preparation and Characterization (SPC) Facility of EMBL, Hamburg. We thank Melanie Scherer and Cedric Hutter for plasmids and protocols. This research was funded by a Heisenberg grant (to H.T.) and the Cluster of Excellence 'Advanced Imaging of Matter' of the Deutsche Forschungsgemeinschaft, EXC 2056, project ID 390715994.

AUTHOR CONTRIBUTIONS

Conceptualization, I.J. and H.T.; methodology, I.J., C.E.B., and H.T.; investigation, L.S., A.G., L.V.S., D.D.V., C.E.B., M.A.S., H.T., and I.J.; writing – original draft, I.J. and H.T.; writing – review and editing, all

authors; funding acquisition, L.V.S., M.A.S., and H.T.; supervision, H.T. and I.J.

DECLARATION OF INTERESTS

The authors declare no competing interests.

SUPPLEMENTAL INFORMATION

Supporting material can be found online at https://doi.org/10.1016/j.bpj.2026.04.016.

REFERENCES

1. Alam, A., and K. P. Locher. 2023. Structure and Mechanism of Human ABC Transporters. *Annu. Rev. Biophys.* 52:275.
2. Dean, M., A. Rzhetsky, and R. Allikmets. 2001. The human ATP-binding cassette (ABC) transporter superfamily. *Genome Res.* 11:1156–1166.
3. Locher, K. P. 2016. Mechanistic diversity in ATP-binding cassette (ABC) transporters. *Nat. Struct. Mol. Biol.* 23:487–493.
4. Cui, J., and A. L. Davidson. 2011. ABC solute importers in bacteria. *Essays Biochem.* 50:85–99.
5. Davidson, A. L., E. Dassa, …, J. Chen. 2008. Structure, function, and evolution of bacterial ATP-binding cassette systems. *Microbiol. Mol. Biol. Rev.* 72:317–364.
6. Higgins, C. F. 1995. The ABC of channel regulation. *Cell.* 82:693–696.
7. Schmitt, L., and R. Tampé. 2002. Structure and mechanism of ABC transporters. *Curr. Opin. Struct. Biol.* 12:754–760.
8. Thomas, C., and R. Tampé. 2020. Structural and Mechanistic Principles of ABC Transporters. *Annu. Rev. Biochem.* 89:605–636.
9. Rees, D. C., E. Johnson, and O. Lewinson. 2009. ABC transporters: the power to change. *Nat. Rev. Mol. Cell Biol.* 10:218–227.
10. Higgins, C. F., and K. J. Linton. 2004. The ATP switch model for ABC transporters. *Nat. Struct. Mol. Biol.* 11:918–926.
11. Janas, E., M. Hofacker, …, R. Tampé. 2003. The ATP hydrolysis cycle of the nucleotide-binding domain of the mitochondrial ATP-binding cassette transporter Mdl1p. *J. Biol. Chem.* 278:26862–26869.
12. Jardetzky, O. 1966. Simple allosteric model for membrane pumps. *Nature.* 211:969–970.
13. Lewinson, O., C. Orelle, and M. A. Seeger. 2020. Structures of ABC transporters: handle with care. *FEBS Lett.* 594:3799–3814.
14. Orelle, C., L. Schmitt, and J. M. Jault. 2023. Waste or die: The price to pay to stay alive. *Trends Microbiol.* 31:233–241.
15. Hofmann, S., D. Januliene, …, A. Moeller. 2019. Conformation space of a heterodimeric ABC exporter under turnover conditions. *Nature.* 571:580–583.
16. Nocker, C., M. Pečak, …, R. Tampé. 2026. Single-molecule dynamics reveal ATP binding alone powers substrate translocation by an ABC transporter. *Nat. Commun.* 17:3038.
17. Stefan, E., S. Hofmann, and R. Tampé. 2020. A single power stroke by ATP binding drives substrate translocation in a heterodimeric ABC transporter. *eLife.* 9:e55943.
18. Kehlenbeck, D. M., D. A. K. Traore, …, H. Tidow. 2022. Cryo-EM structure of MsbA in saposin-lipid nanoparticles (Salipro) provides insights into nucleotide coordination. *FEBS J.* 289:2959–2970.
19. Li, P., A. L. Hendricks, …, P. Gourdon. 2022. Structures of Atm1 provide insight into [2Fe-2S] cluster export from mitochondria. *Nat. Commun.* 13:4339.
20. Lin, D. Y. w., S. Huang, and J. Chen. 2015. Crystal structures of a polypeptide processing and secretion transporter. *Nature.* 523:425–430.
21. Mi, W., Y. Li, …, M. Liao. 2017. Structural basis of MsbA-mediated lipopolysaccharide transport. *Nature.* 549:233–237.
22. Thomas, C., and R. Tampé. 2018. Multifaceted structures and mechanisms of ABC transport systems in health and disease. *Curr. Opin. Struct. Biol.* 51:116–128.
23. Carrillo, V. H. P., M. Di Cesare, …, U. A. Hellmich. 2025. Bidirectional communication between nucleotide and substrate binding sites in a type IV multidrug ABC transporter. *Nat. Commun.* 16:9921.
24. Stefan, E., R. Obexer, …, R. Tampé. 2021. De novo macrocyclic peptides dissect energy coupling of a heterodimeric ABC transporter by multimode allosteric inhibition. *eLife.* 10:e67732.
25. Procko, E., M. L. O'Mara, …, R. Gaudet. 2009. The mechanism of ABC transporters: general lessons from structural and functional studies of an antigenic peptide transporter. *FASEB J.* 23:1287–1302.
26. Hohl, M., C. Briand, …, M. A. Seeger. 2012. Crystal structure of a heterodimeric ABC transporter in its inward-facing conformation. *Nat. Struct. Mol. Biol.* 19:395–402.
27. Hohl, M., L. M. Hürlimann, …, M. A. Seeger. 2014. Structural basis for allosteric cross-talk between the asymmetric nucleotide binding sites of a heterodimeric ABC exporter. *Proc. Natl. Acad. Sci. USA.* 111:11025–11030.
28. Hutter, C. A. J., M. H. Timachi, …, M. A. Seeger. 2019. The extracellular gate shapes the energy profile of an ABC exporter. *Nat. Commun.* 10:2260.
29. Levantino, M., B. A. Yorke, …, A. R. Pearson. 2015. Using synchrotrons and XFELs for time-resolved X-ray crystallography and solution scattering experiments on biomolecules. *Curr. Opin. Struct. Biol.* 35:41–48.
30. Akiyama, S., S. Takahashi, …, T. Fujisawa. 2002. Conformational landscape of cytochrome c folding studied by microsecond-resolved small-angle x-ray scattering. *Proc. Natl. Acad. Sci. USA.* 99:1329–1334.
31. Arai, M., E. Kondrashkina, …, O. Bilsel. 2007. Microsecond hydrophobic collapse in the folding of Escherichia coli dihydrofolate reductase, an alpha/beta-type protein. *J. Mol. Biol.* 368:219–229.
32. Konuma, T., T. Kimura, …, S. Takahashi. 2011. Time-resolved small-angle X-ray scattering study of the folding dynamics of barnase. *J. Mol. Biol.* 405:1284–1294.
33. West, J. M., J. Xia, …, E. R. Kantrowitz. 2008. Time evolution of the quaternary structure of Escherichia coli aspartate transcarbamoylase upon reaction with the natural substrates and a slow, tight-binding inhibitor. *J. Mol. Biol.* 384:206–218.
34. Josts, I., Y. Gao, …, H. Tidow. 2020. Structural Kinetics of MsbA Investigated by Stopped-Flow Time-Resolved Small-Angle X-Ray Scattering. *Structure.* 28:348.
35. Blanchet, C. E., A. Spilotros, …, D. I. Svergun. 2015. Versatile sample environments and automation for biological solution X-ray scattering experiments at the P12 beamline (PETRA III, DESY). *J. Appl. Crystallogr.* 48:431–443.
36. Franke, D., C. M. Jeffries, and D. I. Svergun. 2015. Correlation Map, a goodness-of-fit test for one-dimensional X-ray scattering spectra. *Nat. Methods.* 12:419–422.
37. Franke, D., M. V. Petoukhov, …, D. I. Svergun. 2017. ATSAS 2.8: a comprehensive data analysis suite for small-angle scattering from macromolecular solutions. *J. Appl. Crystallogr.* 50:1212–1225.
38. Webb, B., and A. Sali. 2021. Protein Structure Modeling with MODELLER. *Methods Mol. Biol.* 2199:239–255.
39. Cheng, X., S. Jo, …, W. Im. 2013. CHARMM-GUI micelle builder for pure/mixed micelle and protein/micelle complex systems. *J. Chem. Inf. Model.* 53:2171–2180.
40. Svergun, D., C. Barberato, and M. H. J. Koch. 1995. CRYSOL - a Program to Evaluate X-ray Solution Scattering of Biological Macromolecules from Atomic Coordinates. *J. Appl. Crystallogr.* 28:768–773.
41. Baginski, E. S., E. Epstein, and B. Zak. 1975. Review of phosphate methodologies. *Ann. Clin. Lab. Sci.* 5:399–416.
42. Chifflet, S., A. Torriglia, …, S. Tolosa. 1988. A method for the determination of inorganic phosphate in the presence of labile organic phosphate and high concentrations of protein: application to lens ATPases. *Anal. Biochem.* 168:1–4.

43. Kehlenbeck, D. M., I. Josts, …, H. Tidow. 2019. Comparison of lipidic carrier systems for integral membrane proteins - MsbA as case study. *Biol. Chem.* 400:1509–1518.

44. Göddeke, H., and L. V. Schäfer. 2020. Capturing Substrate Translocation in an ABC Exporter at the Atomic Level. *J. Am. Chem. Soc.* 142:12791–12801.

45. Göddeke, H., M. H. Timachi, …, L. V. Schäfer. 2018. Atomistic Mechanism of Large-Scale Conformational Transition in a Heterodimeric ABC Exporter. *J. Am. Chem. Soc.* 140:4543–4551.

46. Mishra, S., B. Verhalen, …, H. S. Mchaourab. 2014. Conformational dynamics of the nucleotide binding domains and the power stroke of a heterodimeric ABC transporter. *eLife.* 3:e02740.

47. Verhalen, B., R. Dastvan, …, H. S. Mchaourab. 2017. Energy transduction and alternating access of the mammalian ABC transporter P-glycoprotein. *Nature.* 543:738–741.

48. Timachi, M. H., C. A. Hutter, …, E. Bordignon. 2017. Exploring conformational equilibria of a heterodimeric ABC transporter. *eLife.* 6:e20236.

49. Galazzo, L., G. Meier, …, E. Bordignon. 2020. Spin-labeled nanobodies as protein conformational reporters for electron paramagnetic resonance in cellular membranes. *Proc. Natl. Acad. Sci. USA.* 117:2441–2448.

Biophysical Journal, Volume *125*

Supplemental information

Capturing transient states of heterodimeric ABC transporter TM287/288 by time-resolved small-angle X-ray scattering

Lea Schröder, Dario De Vecchis, Andrey Gruzinov, Lars V. Schäfer, Clement E. Blanchet, Markus A. Seeger, Henning Tidow, and Inokentijs Josts

Supplementary Information for:

Capturing transient states of heterodimeric ABC transporter TM287/288 by Time-Resolved Small-Angle X-ray Scattering

Lea Schröder[1,2], Dario De Vecchis[3,7], Andrey Gruzinov[4], Lars V. Schäfer[3], Clement E. Blanchet[4], Markus A. Seeger[5], Henning Tidow[1,2]*, and Inokentijs Josts[1,2,6]*

[1] The Hamburg Advanced Research Centre for Bioorganic Chemistry (HARBOR), Luruper Chaussee 149, D-22761 Hamburg, Germany

[2] Department of Chemistry, University of Hamburg, Luruper Chaussee 149, D-22761 Hamburg, Germany

[3] Center for Theoretical Chemistry, Ruhr University Bochum, Universitätsstr. 150, D-44801 Bochum, Germany

[4] European Molecular Biology Laboratory Hamburg Outstation c/o DESY, Notkestrasse 85, D-22607 Hamburg, Germany

[5] Institute of Medical Microbiology, University of Zurich, Zürich, Switzerland

[6] Newcastle University, Faculty of Medical Sciences, Newcastle University Biosciences Institute (NUBI), Framlington Place, Newcastle upon Tyne NE2 4HH, UK

[7] Present address: Univ. Grenoble Alpes, CNRS, CEA, IBS, France

* Corresponding authors:

Inokentijs Josts
Newcastle University, Faculty of Medical Sciences, Newcastle University Biosciences Institute (NUBI), Framlington Place, Newcastle upon Tyne NE2 4HH, UK
e-mail: Kesha.Josts@newcastle.ac.uk

Henning Tidow
University of Hamburg, The Hamburg Advanced Research Centre for Bioorganic Chemistry (HARBOR), Luruper Chaussee 149, D-22761 Hamburg, Germany
e-mail: henning.tidow@uni-hamburg.de

Supplementary figures

Suppl. Figure S1

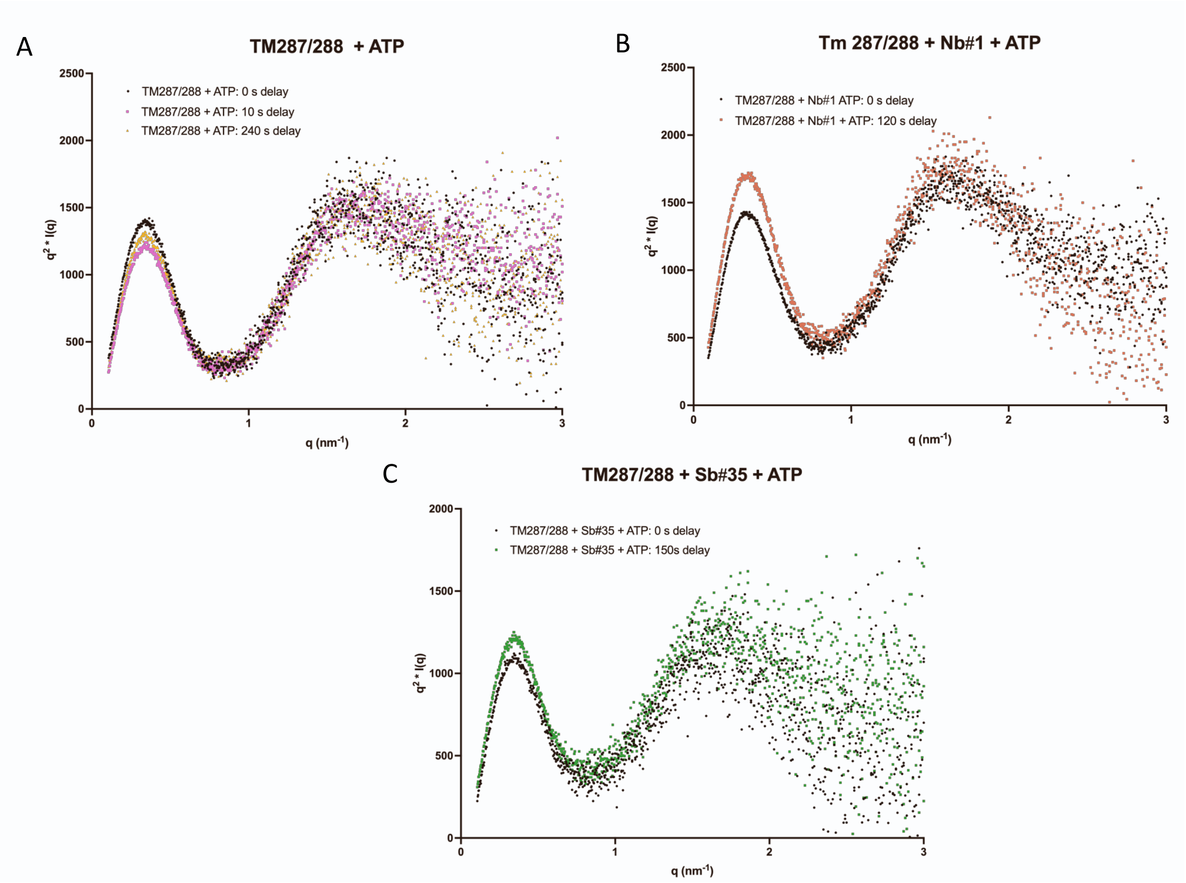

Kratky plots of representative experimental SAXS curves at selected time points. A) Three time points of TM287/288 binding ATP-Mg^{2+} representing evolution of conformational changes in the sample. B) Two time points showing changes in scattering of TM287/288 upon binding ATP and Nb#1. C) Two time points showing changes in scattering of TM287/288 upon binding ATP and Sb#35.

Suppl. Figure S2

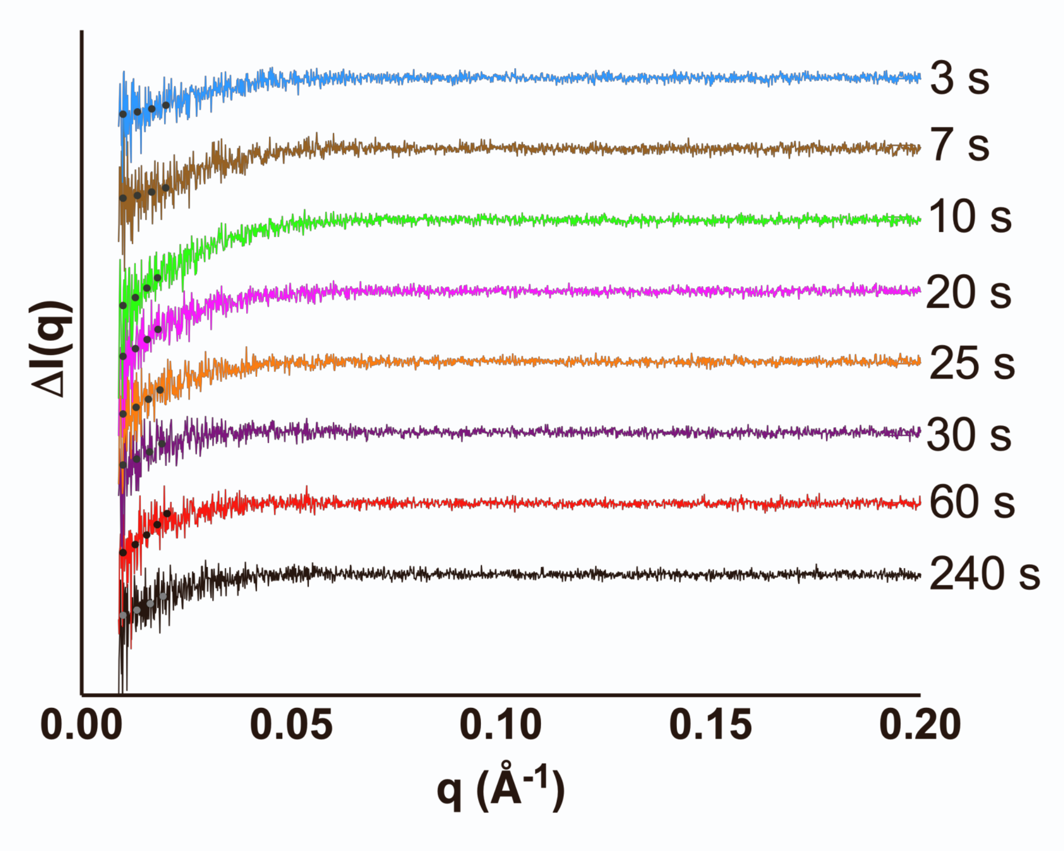

Time-resolved scattering difference curves show conformational changes in TM287/288 upon ATP binding. Stacked ΔI(q) curves showing changes in scattering signal over reaction time. Dotted lines emphasise changes in low-q region where Guinier fits were calculated.

Suppl. Figure S3

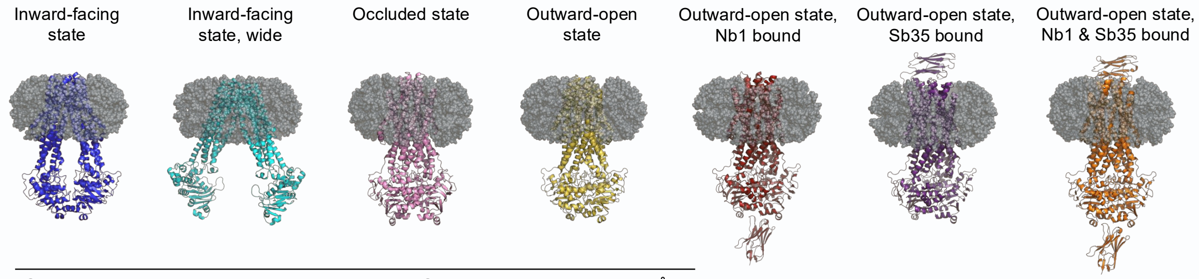

State	Source (PDB)	R_g [Å]
Inward-facing	4Q4A	44.2
Inward-facing, wide	6BL6	47.9
Occluded	modelled from: 4S0F, 7PR1, 6RAI	44.0
Outward-open	6QUZ	45.4
Outward-open, Nb1 bound	modelled from: 6QV1	48.5
Outward-open, Sb35 bound	modelled from: 6QUZ	46.0
Outward-open, Nb1 & Sb35 bound	modelled from: 6QV1, 6QUZ	48.9

Illustration of different conformational states of TM287/288 in detergent (DDM) micelles with R_g values indicated. PDB codes of structures or templates for models are given in the table. inward-facing state, blue / wide inward-facing apo state, cyan / occluded state, pink / outward-open state, yellow / outward-open, Nb1 bound state, red / outward-open Sb35-bond state, violet / outward-open Nb1 and Sb35-bound state, orange. Detergent micelles were added using CHARMM-GUI micelle builder (Cheng, Jo et al. 2013), and R_g values were calculated using CRYSOL (Svergun, Barberato et al. 1995).

Suppl. Figure S4

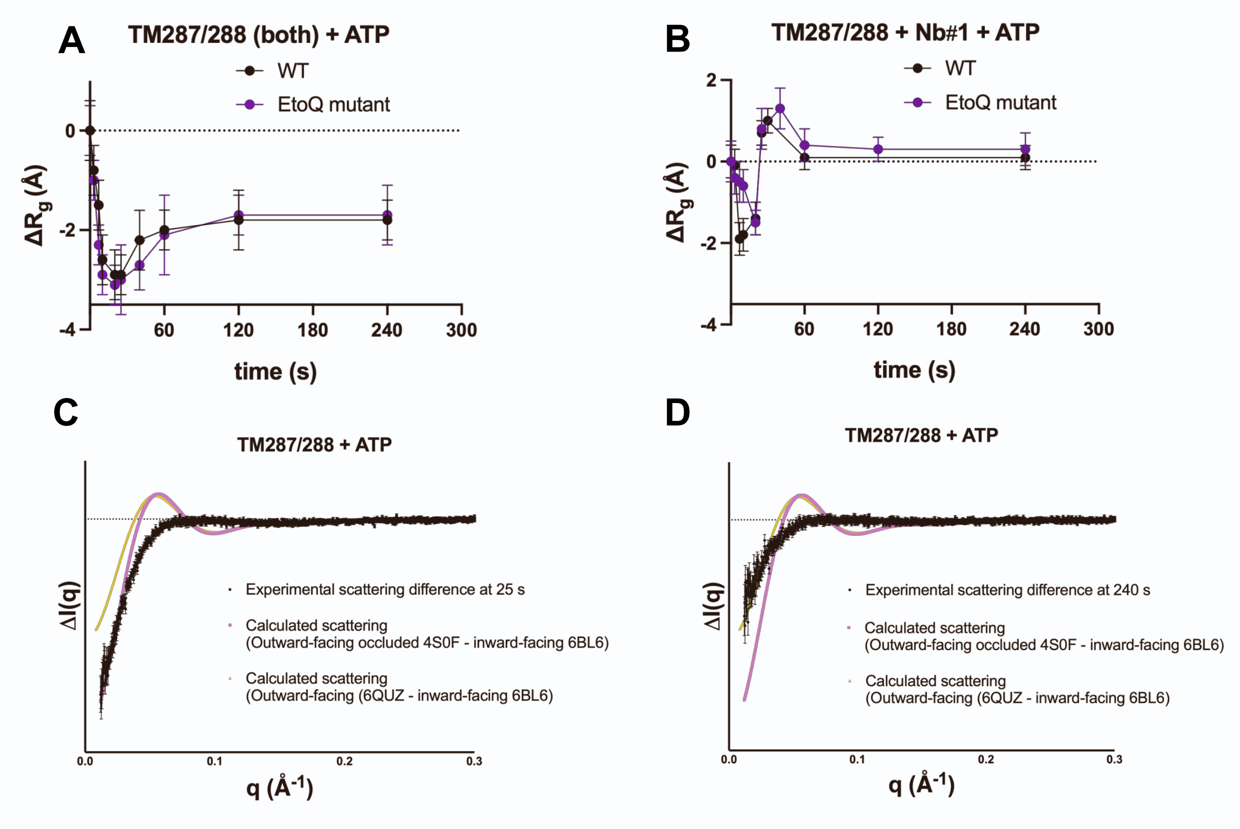

SF-TR-SAXS comparison of wt TM287/288 and its E/Q-mutant (E517Q). A) Apo proteins mixed with ATP-Mg^{2+}. B) TM287/288 variants premixed with Nb#1 before SF-mixed with ATP-Mg^{2+}. C) and D) Comparison of the 25 s time point (where the occluded state is thought to populate) and 240 s time point (where outward-open state is thought to populate) in our kinetic reaction with two calculated models of TM287/288. The χ^2 values for the fit between 25 s and Occ model is 13.8 versus 26.7 for OF model. At 240 s the χ^2 for Occ is 10 but 3.9 for OF model. The 240 s difference dataset is noisier and larger experimental uncertainties lead to lower χ^2 values.

Suppl. Figure S5

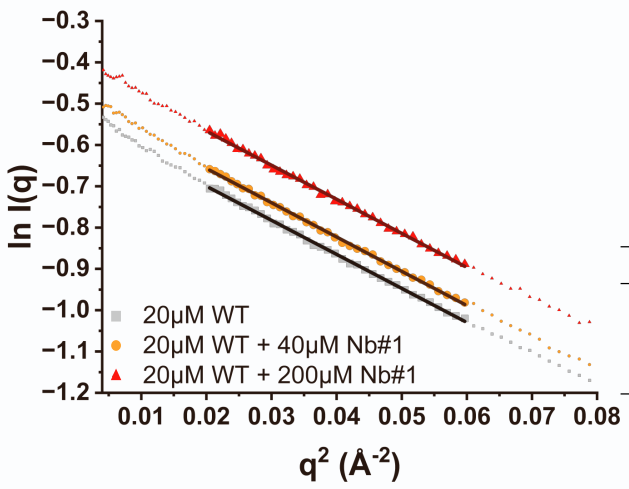

sample	slope Guinier plot	R_g [Å]
TM287/288 WT	-8.25 ± 0.05	49.7 ± 0.2
TM287/288 WT + 2x Nb#1	-8.30 ± 0.05	49.9 ± 0.2
TM287/288 WT + 10x Nb#1	-8.25 ± 0.07	49.7 ± 0.3

Guinier analysis of apo TM287/288 in absence and presence of Nb#1 indicate that Nb#1 is not binding to TM287/288 in the absence of Mg^{2+}-ATP.

Suppl. Figure S6

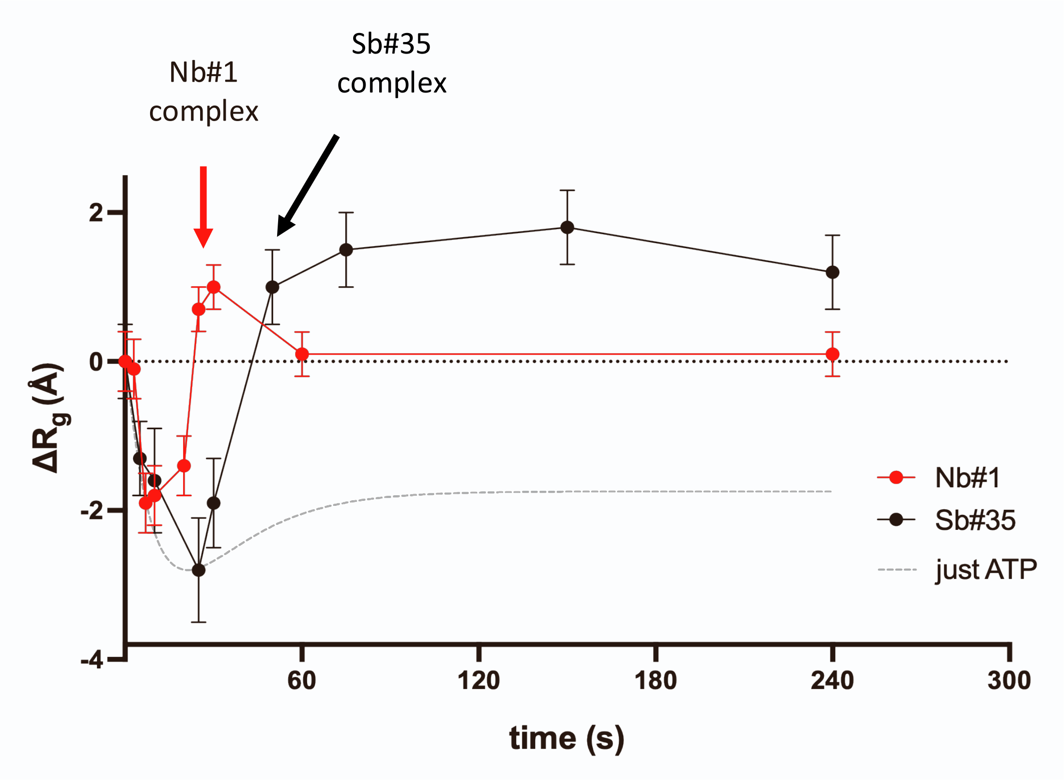

SF-TR-SAXS comparison of TM287/288 in complex with Nb#1 and Sb#35. SF-TR-SAXS data acquired for TM287/288 premixed with Nb#1 (A) or Sb#35 (B) before SF-mixed with ATP-Mg^{2+}. Nb#1 binds to the NBDs of TM287/288 in the occluded state while Sb#35 binds to the extracellular side only in the OF state.

Suppl. Figure S7

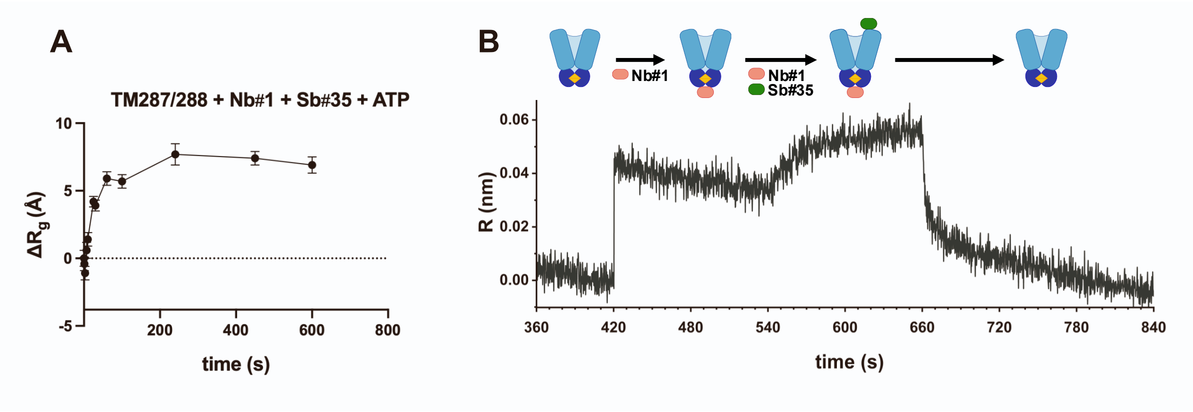

Simultaneous binding of both single-domain antibodies to TM287/288. A) SF-TR-SAXS data acquired for TM287/288 premixed with both Nb#1 and Sb#35 before SF-mixed with ATP-Mg^{2+}. B) BLI measurements showing association of Nb#1 and Sb#35 followed by their dissociation.

Suppl. Table 1

	K_m	v_{max}	k_{cat}
	mM	*$nmol_{ATP}$/min/$mg_{TM287/288}$*	*min^{-1}*
**wt**	0.059 ± 0.008	67.7 ± 1.5	9.02 ± 0.20
**wt 2x Nb1**	0.039 ± 0.005	45.6 ± 0.8	6.07 ± 0.11
**wt 10x Nb1**	0.066 ± 0.008	41.4 ± 0.9	5.52 ± 0.12
**wt 2x Sb35**	0.041 ± 0.005	49.5 ± 0.9	6.61 ± 0.12
**wt 10x Sb35**	0.048 ± 0.008	36.5 ± 0.9	4.87 ± 0.12
**E517Q**	0.038 ± 0.018	12.5 ± 0.8	1.67 ± 0.11

Suppl. Table S1: ATPase activity assays. Activity assays for TM287/288 were performed using the Baginski method at room temperature (Baginski, Epstein et al. 1975, Chifflet, Torriglia et al. 1988). K_m and k_{cat} were calculated from fitting the experimental data according to Michaelis-Menten. Standard errors were obtained from triplicate measurements.

References

Baginski, E. S., E. Epstein and B. Zak (1975). "Review of phosphate methodologies." Ann Clin Lab Sci **5**(5): 399-416.

Cheng, X., S. Jo, H. S. Lee, J. B. Klauda and W. Im (2013). "CHARMM-GUI micelle builder for pure/mixed micelle and protein/micelle complex systems." J Chem Inf Model **53**(8): 2171-2180.

Chifflet, S., A. Torriglia, R. Chiesa and S. Tolosa (1988). "A method for the determination of inorganic phosphate in the presence of labile organic phosphate and high concentrations of protein: application to lens ATPases." Anal Biochem **168**(1): 1-4.

Svergun, D. I., C. Barberato and M. H. J. Koch (1995). "CRYSOL - a Program to Evaluate X-ray Solution Scattering of Biological Macromolecules from Atomic Coordinates." J. Appl. Crystallogr. **28**: 768-773.
